# Effect of Intensified Fermentation with *Wickerhamomyces anomalus* on Fungal Community Structure of Fermented Grains and Flavor Compounds of *Xiaoqu Baijiu*

**DOI:** 10.3390/foods14193365

**Published:** 2025-09-29

**Authors:** Jie Deng, Bo Zeng, Chunhui Wei, Zhiguo Huang

**Affiliations:** Brewing Science and Technology Key Laboratory of Sichuan Province, Sichuan University of Science & Engineering, Yibin 644000, China

**Keywords:** *Xiaoqu Baijiu*, Fungal community structure, *Wickerhamomyces*, Bioaugmentation, Flavor compounds

## Abstract

In order to explore the effect of *Wickerhamomyces* on the production of flavor compounds in *Xiaoqu Baijiu* (*XQBJ*), this study examined the correlation between the fungal communities in *Xiaoqu* and ester compounds. It was hypothesized that *Wickerhamomyces* contributes to the aroma of Xiaoqu. Intensified fermentation methods were used to validate the role of *Wickerhamomyces anomalus* in *XQBJ* brewing. Compared to traditional *Xiaoqu*, intensified fermentation significantly increased the acidity of fermented grains and starch utilization rate (*p* < 0.05). The fungal communities in fermented grains were analyzed by high-throughput sequencing technology. The dominant fungi in both control and test groups were *Saccharomyces*, *Cyberlindnera*, *Rhizopus*, and *Meyerozyma*, with *Wickerhamomyces* replacing *Saccharomymycopsis* in the enhanced group. Flavor compounds in the fermentation experiments were analyzed, revealing that ethyl acetate content in *XQBJ* increased by 949.94 mg/L in the test group, while isoamyl alcohol decreased by 23.62 mg/L and isobutanol decreased by 34.81 mg/L. Functional prediction analysis using PICRUSt2 confirmed a higher relative abundance of enzymes involved in ethyl acetate metabolism and a lower relative abundance of enzymes involved in higher alcohol metabolism in the test group. These findings demonstrate that *Wickerhamomyces* enhances ethyl acetate production and reduces higher alcohols during *XQBJ* brewing, offering a theoretical foundation for enhancing the quality of *XQBJ*.

## 1. Introduction

The industry of *Baijiu* is rooted in traditional brewing practices. However, with modern technological advancements and scientific research, *Baijiu* production methods have continually evolved and improved [1]. *Xiaoqu Baijiu* (*XQBJ*) has a rich history and is distinguished by its crisp and pure aroma, mellow and balanced flavor, and invigorating aftertaste [2,3]. *XQBJ* is made from sorghum as the raw material, with *Xiaoqu* as the saccharifying and fermenting agent, and produced through saccharification, fermentation, and distillation processes (Figure S1A). *XQBJ* has a simpler brewing process compared to other types of Baijiu. Additionally, its fermentation period is shorter, the alcohol yield is higher, and alcohols and esters are the main aroma compounds. *XQBJ* does, however, have several drawbacks, including a thin body, a narrow range of flavors, high concentrations of fusel alcohols, and low levels of ethyl acetate [3]. At present, the main focus of research is on increasing the content of key flavor compounds in *XQBJ* and improving its quality.

Enhancing fermentation with high-quality microbial strains is an effective method for significantly improving the flavor and quality of *Baijiu*. Studies have shown that incorporating *Rhizopus Fuqu* can increase the lactic acid ethyl ester content in Jiang-flavor *Baijiu* [4]. Intensified fermentation using aroma-producing functional microbes in *Fuqu* has elevated the concentrations of acids, alcohols, and esters in fermented grains. At the same time, previous studies also proved that it was the function of the microbial in *Fuqu* to produce the flavor, rather than the effect of the bran [3,5,6]. This indicates that incorporating functional microbes into *Fuqu* for fortified fermentation is a successful strategy. *Wickerhamomyces* demonstrate excellent stability and fermentation efficiency, offering promising potential for streamlining fermentation procedures and boosting the flavor of fermented beverages [7]. Currently, *Wickerhamomyces* is extensively utilized in the fermentation industry. Employing *Wickerhamomyces* in Chinese Te-flavor *Baijiu* brewing markedly augmented the ethyl esters content [8,9]. Using *Saccharomyces* and *Wickerhamomyces* in the fermentation of rice wine significantly increased the quantities of flavor compounds, including phenylethyl alcohol, isobutanol, and hexanol [7,9,10,11]. Mixing culture with *Wickerhamomyces* could improve flavor metabolism of *Saccharomyces* in *Baijiu*-making [12]. Overall, *Wickerhamomyces* contributes positively to enhancing the flavor of alcoholic beverages. Nevertheless, there is a lack of research on the application of *Wickerhamomyces* in *XQBJ*, and the mechanism of its aroma production is not well understood. Further research is needed to determine if incorporating it into *Fuqu* as a medium can enhance the quality of *XQBJ*.

Our study hypothesizes that *Wickerhamomyces* could potentially enhance the flavor of *XQBJ* by examining the correlation between the fungal community structure of *Xiaoqu* and ester compounds. By incorporating *Wickerhamomyces* into the fermentation of *Xiaoqu*, using *Fuqu* as the medium, we aimed to investigate the effects of this fortified fermentation on both the fermentation environment and alcohol yield. The effects of fortified fermentation on the flavor compounds of *XQBJ* were examined using gas chromatography. Additionally, high-throughput sequencing technology was employed to analyze how fortified fermentation disrupted the fungal community structure in fermented grains. Furthermore, PICRUSt2 marker gene sequences were utilized to predict the types and abundances of enzymes associated with distinct compounds. This study investigated the feasibility of using *Wickerhamomyces* with *Fuqu* as the medium for the fermentation of *XQBJ*, aiming to enhance the quality of *XQBJ*.

## 2. Materials and Methods

### 2.1. Fermentation of XQBJ

The fermentation substrate used was commercially available Japonica sorghum, along with its husk. Three types of *Xiaoqu* (XQ1, XQ2, XQ3) were purchased from local markets and their microbial communities were analyzed. A simulation experiment was conducted based on the brewing process of XQBJ, and the production process is shown in Figure S1A. The sorghum was soaked in boiling water for 24 h. It was then cooked twice and pasteurized to ensure that the moisture content of the cooked grain was above 50%. Once the grains had cooled, 5% (5 g/100 g) of steamed husk was added, and *Xiaoqu* was added for saccharification 24 h later (0.6%, 0.6 g/100 g). The saccharified sorghum was fermented using a mixed distillation method for 7 days. This process was carried out in a 5L fermentation tank. Following the completion of fermentation, the fermented grains were distilled to obtain *Baijiu* samples. Each experimental group consisted of three replicates.

### 2.2. Production of Wickerhamomyces Fuqu (WF)

*Wickerhamomyces anomalus* was isolated from *Xiaoqu*, then it was stored in Brewing Science and Technology Key Laboratory of *Sichuan* Province. Square stainless steel boxes were utilized as the container, adhering to the traditional *Fuqu* production technique (Figure S1B). An amount of 20 kg of bran was weighed and water was added to achieve a moisture content of 55%. The mixture was sealed with four layers of gauze and sterilized at 100 °C for 20 min. In an aseptic centrifuge tube, 400 mL of seed liquid was collected and centrifuged at 500 rpm, and the supernatant was discarded. The cells were then rinsed with 400 mL of sterile water inoculated into the sterilized bran. After thorough mixing and covering with gauze, it was cultivated in an incubator at 30 °C for 36 h. Upon the completion of cultivation, it was dried in an oven at 30 °C and stored for future use.

### 2.3. Preparation for Intensive Fermentation

The fermentation experiment was carried out according to the production process of XQBJ (Figure S1A). In the experimental group (QH), sorghum underwent soaking, cooking, and cooling before the addition of WF (0.1%, 0.1 g/100 g) and XQ2 (0.6%, 0.6 g/100 g). In the control group (DZ), only XQ2 (0.6%, 0.6 g/100 g) was added. This fermentation process was identical to that described in Section 2.1. Cooked sorghum (ZL), saccharified fermented grains (TH), and fermented grains collected on days 1, 3, 5, and 7 were subjected to analysis. After thorough mixing, the samples were stored at −20 °C for further analysis.

### 2.4. Detection of Physicochemical Indicators of Fermented Grains

The moisture content was assessed using the gravimetric method. The acidity of the fermented grains was measured through acid–base titration. Quantification of reducing sugars and starch was carried out using Fehling’s reagent method, following the procedure outlined by Deng [13].

### 2.5. Detection of Flavor Compounds in XQBJ

The headspace vial was placed in a water bath set at 50 °C for 20 min to achieve thermal equilibrium. Subsequently, a 50/30 DVB/CAR/PDMS extraction head was inserted for a 30-min adsorption period, and the extraction was analyzed by GC-MS (8890 GC, 5977C Mass, Agilent Technologies, Santa Clara, CA, USA). Chromatographic analysis was conducted using a DB-Wax capillary column (60 m × 0.25 mm × 0.25 µm), and high-purity helium was used as the carrier gas. The flavor compounds in *Baijiu* samples were quantified using hydrogen flame gas chromatography (GC-FID) ( 8890GC, Agilent Technologies, Santa Clara, CA, USA) and GC-MS/MS (7890 GC, 7000D Mass, Agilent Technologies, Santa Clara, CA, USA). The chromatographic column used was LZP-950.2 (50 m× 0.32 mm × 1 µm), and high-purity nitrogen was used as the carrier gas. The specific methods are referred to as follows by Deng [14].

### 2.6. Calculation of Odor Activity Value (OAV) and Flavor Dilution (FD)

The odor activity value (OAV) for each flavor component was measured using the aroma intensity method. A higher OAV signifies that the flavor component has a stronger aroma intensity in Baijiu, thereby exerting a greater influence on the overall aroma profile of the beverage. The calculation formula is as follows:

$$\mathrm{OAV}=\frac{\mathrm{xi}}{\mathrm{OTi}}\times 10^{6}$$

where xi is the concentration of the flavor component in g/L and OTi is the odor threshold value of the flavor component in μg/L.

The aroma-active compounds were identified by GC-O (8890 GC, Agilent Technologies, Santa Clara, CA, USA, ODP C300, Gerstel, Mülheim, Germany). The shunt ratio between MS and ODP was 1:1, with the ODP transmission line and olfactory port temperatures set to 250 °C and 200 °C, respectively. The flavor dilution (FD) of the aroma-active substances is calculated by aroma extraction dilution analysis (AEDA), and the specific method was performed as follows by Deng [14].

### 2.7. DNA Extraction and High-Throughput Sequencing

Ten grams of samples were weighed and mixed with 25 mL of sterilized and cooled 0.1 mol/L PBS buffer, along with sterile glass beads. The mixture was vortexed for 30 min and then washed thrice with PBS buffer. The supernatant was collected by centrifugation (300× *g*, 5 min) and all supernatants were combined to obtain the precipitate, which was then washed thrice with PBS (9000× *g*, 3 min). DNA extraction was carried out using the phenol–chloroform method. The density and quality of the extracted DNA were assessed using a NanoDrop ND-2000 spectrophotometer (Illumina, San Diego, CA, USA) and 1% agarose gel electrophoresis. For fungal amplification, primers ITS1F (5′-CTTGGTCATTTAGAGGAAGTAA-3′) and ITS4R (5′-TCCTCCGCTTATTGATATGC-3′) were used. PCR was conducted in triplicate with a 20 μL mixture using a MyCycler Thermal (Bio-Rad, Stateof, CA, USA), following the method described by Liu [12]. Subsequent library preparation for next-generation sequencing and high-throughput sequencing was carried out at Shanghai Majorbio Biopharm Technology Co., Ltd. (Shanghai, China).

Quality control and assembly of the raw sequenced data were performed using Trimmomatic0.36 software (https://cloud.majorbio.com/, accessed on 11 April 2024) and FLASH software (https://cloud.majorbio.com/, accessed on 11 April 2024), respectively. Operational taxonomic units (OTUs) were clustered based on 97% similarity, with single sequence removal and chimera removal conducted using UPARSE software (version 7.1 http://drive5.com/uparse/, accessed on 15 April 2024). Each sequence was taxonomically annotated using the RDP classifier and compared with the database.

### 2.8. Statistical Analysis

Statistical Product and Service Solutions (SPSS) was employed for significant difference analysis. Origin2018 software analyzed changes in physicochemical indicators and microorganisms in fermented grains. R4.2.2 software was used to create heat maps for visualization and conduct redundancy analysis. Using the KEGG (Kyoto Encyclopedia of Genes and Genomes) database and the PICRUSt tool, online functional prediction was conducted based on OTU classification. ASV sequences were aligned with reference sequences and placed into a reference tree to infer gene families’ copy numbers and predict gene content. This data was integrated with MinPath to determine gene family abundance in each sample. The gene family information was then compared with the KEGG database to obtain functional information and abundance data, which was used to create metabolic pathways and bubble charts based on enzyme abundance.

## 3. Results

### 3.1. The Correlation Between Fungal Community and Flavor Compounds in XQBJ

**Figure 1:**
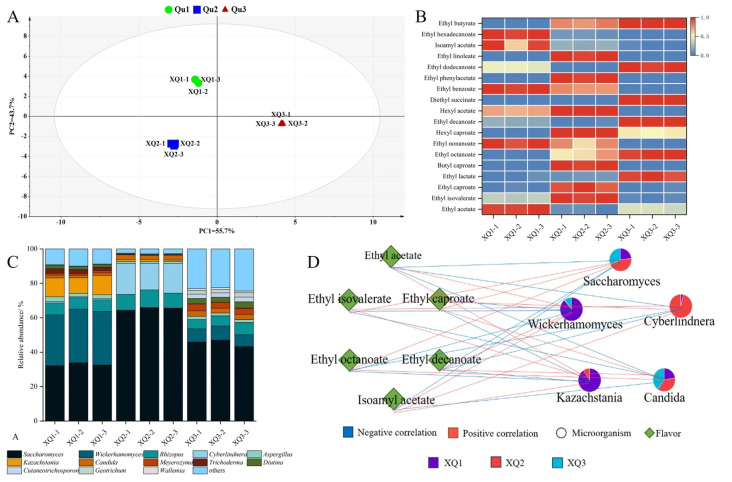
Correlation between the fungal community and key ester compounds in *Xiaoqu Baijiu*. ((**A**) PCA of the flavor compounds in *Xiaoqu Baijiu*. (**B**) The ester compounds heatmap; (**C**) The fungal community structure in *Xiaoqu*; (**D**) The correlation analysis of fungal community and key ester compounds).

Forty-six volatile compounds in nine samples of *XQBJ* were determined. PCA based on volatile compounds results were conducted, and PCA score plots are shown in Figure 1A. The first two principal components contributed to elucidating 99.4% of the total variance; the samples of *XQBJ* were classified into three categories, indicating that there are significant differences in the volatile compounds. Meanwhile, the three samples show significant differences in the composition and content of ester compounds across different *XQBJ* samples (Figure 1B). The OAVs for flavor compounds were calculated, and the flavor compounds with OAV > 1 are presented in Table 1, including ethyl acetate, ethyl isovalerate, ethyl caproate, ethyl decanoate, ethyl octanoate, and isoamyl acetate. Six aroma compounds (OAV > 1) showed fruity or floral odors through aroma activity analysis, and the flavor dilution value (FDs) of ethyl acetate and isoamyl acetate are different among three samples of XQBJ.

The microbial community structure of nine samples of *Xiaoqu* was analyzed using Illumina MiSeq System, and the results were presented using bar charts (Figure 1C). *Saccharomyces*, *Rhizopus*, *Aspergillus*, and *Candida* were the common dominant fungal genera (relative abundance >1%) across the three types of *Xiaoqu*. *Cyberlindnera* was the dominant fungal genus unique to XQ2, contributing to the metabolism of acetate and ethyl esters, thereby increasing the concentration of flavor-active esters [16]. *Trichoderma* was the main genus specific to XQ1, with secondary metabolites including phenolic compounds, ketones, and terpenoids [17]. *Wickerhamomyces* had the highest relative abundance in XQ1, producing various glycosidases such as β-D-glucosidase, β-D-xylosidase, α-L-rhamnosidase, etc. *Wickerhamomyces* contributed to aroma, ester, and alcohol production, significantly enhancing Baijiu’s sensory quality, and making it a crucial functional microorganism in the fermentation process [12]. The correlation between six flavor compounds with OAV > 1 and five dominant fungi was analyzed (Figure 1D). There was a notable correlation between these fungi and ester compounds. Ethyl acetate displayed a negative correlation with *Saccharomyces* and a positive correlation with *Wickerhamomyces*. *Cyberlindnera* was positively correlated with ethyl isovalerate and negatively correlated with ethyl decanoate. Additionally, ethyl caprylate and isoamyl acetate showed positive correlations with *Candida* and *Kazachstania*, respectively.

Therefore, the presence of fungi in *Xiaoqu* could influence the metabolism of ester compounds during the fermentation process of *XQBJ*. Ethyl acetate represented the primary flavor substance in *XQBJ*. Modulating the ethyl acetate content could potentially alter the overall quality of *Baijiu*, with a notable positive correlation observed between this compound and *Wickerhamomyces*. Enhancing the fermentation process with *Wickerhamomyces* in *Xiaoqu* might thus enhance the flavor profile of *XQBJ*.

### 3.2. Effects of WF Intensive Fermentation on Physicochemical Indicators

**Figure 2:**
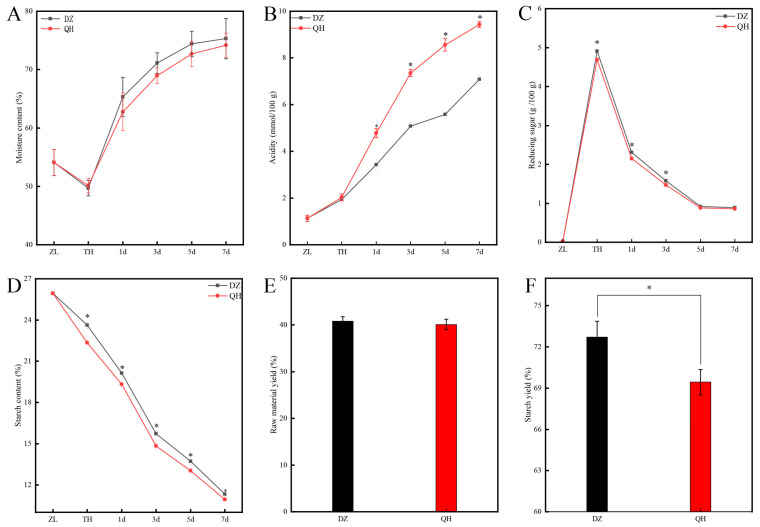
The physicochemical indicators of fermented grains during the fermentation of XQBJ. ((**A**) The content of moisture; (**B**) The content of acidity; (**C**) The content of reducing sugar; (**D**) The content of starch; (**E**) The yield of ethanol from the raw materials; (**F**) The yield of ethanol from starch). *: Indicate significant difference (*p* < 0.05).

Four physicochemical indicators of the fermented grains were tested, including moisture content, acidity, starch, and reducing sugar. The moisture content of cooked sorghum was 54.08%, and the moisture content in the experimental group and the control group during the saccharification process decreased by 4.36% and 3.96%, respectively (Figure 2A). Throughout the fermentation process, the moisture content of fermented grains showed a general increase. There were no significant differences in moisture content between the control and experimental groups at corresponding fermentation stages (*p* > 0.05). The acidity levels showed a tendency to rise with the advancement of fermentation (Figure 2B). There were no significant differences observed between the control and experimental groups in the acidity of fermented grains following cooking and saccharification. The acidity of fermented grains in the experimental group after 1 d was significantly elevated compared to the control group (*p* < 0.05), suggesting that *Wickerhamomyces* metabolized and generated acids during fermentation, thereby increasing the overall acidity of the fermented grains. In Figure 2C, it is evident that the highest levels of reducing sugars occurred at the conclusion of saccharification. Throughout the time points of TH, 1 d, and 3 d, the control group consistently exhibited significantly greater amounts of reducing sugars compared to the experimental group (*p* < 0.05). The starch content exhibited a decline over the fermentation period. However, at TH, 1 d, 3 d, 5 d, and 7 d, the experimental group consistently showed significantly lower starch content compared to the control group (*p* < 0.05) (Figure 2D). Although there was no significant difference in the raw material ethanol yield rate between the control and the experimental group (*p* > 0.05) (Figure 2E), the starch ethanol yield rate in the experimental group was significantly lower than that in the control group (*p* < 0.05) (Figure 2F).

### 3.3. Effect of WF Intensive Fermentation on Flavor Compounds of XQBJ

**Figure 3:**
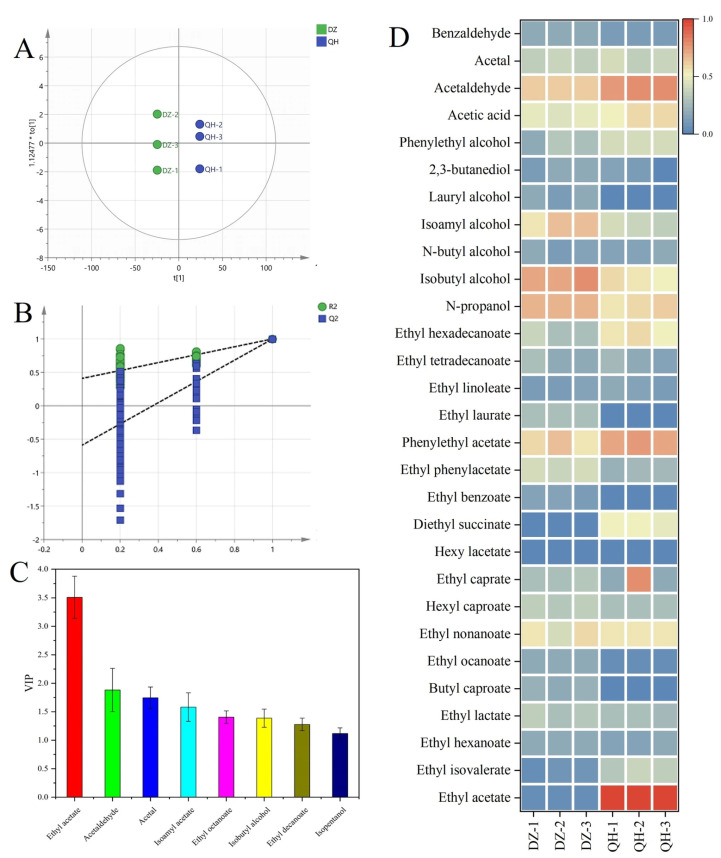
Comparative analysis of flavor compounds between the two groups. ((**A**) Partial least squares-discrimination analysis; (**B**) Permutation test; (**C**) VIP value; (**D**) flavor substance heatmap).

GC-MS was employed to analyze the volatile flavor compounds in *XQBJ*. Overall, 48 volatile compounds were detected in *XQBJ*, which included 19 esters, 11 alcohols, 5 acids, and 13 others. In addition, partial least squares discrimination analysis (PLS-DA) was conducted based on volatile compounds data, and the distinct separation of *XQBJ* samples from the control and test groups on the PLS-DA score plot (Figure 3A). The quality of the model fit was evaluated using a permutation test (*n* = 200), yielding *R*^2^*Y* = 0.48 and *Q*^2^*Y* = −0.59 (Figure 3B). These indices indicate the model explains some variance, the negative *Q^2^Y* value suggests a poor fit of the model. Based on the variable importance in projection (VIP) values (>1), eight volatile compounds were identified as markers, comprising four esters (ethyl acetate, isoamyl acetate, ethyl caprylate, ethyl decanoate), two aldehydes (acetaldehyde, acetal), and two alcohols (isobutanol and isoamyl alcohol) (Figure 3C). The data was normalized to visualize the concentration of volatile compounds in the samples (Figure 3D). Importantly, the experimental group showed significantly higher levels of ethyl acetate compared to the control group. Ethyl acetate was identified as the primary contributor in the discriminant model and is a key aromatic component of *XQBJ*, influencing its quality and character. Based on these findings, we tentatively concluded that WF-intensive fermentation has positively influenced the quality of *XQBJ*.

**Figure 4:**
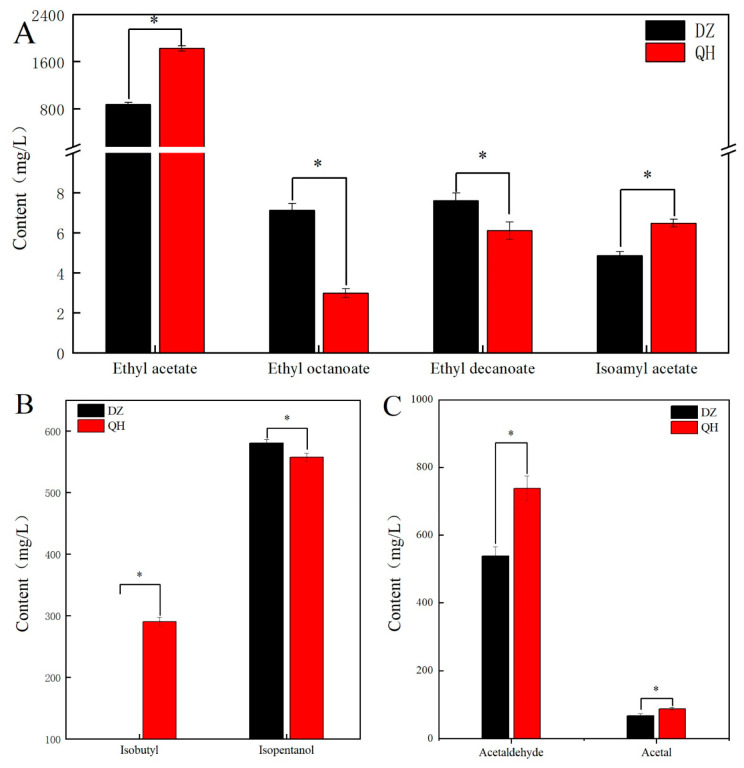
Comparison of flavor compounds between control and test groups. ((**A**) The content of esters; (**B**) The content of alcohol; (**C**) The content of aldehydes). *: Indicate significant difference (*p* < 0.05).

Due to the poor fit of the model, in order to verify the selected differential compounds, the flavor compounds identified as having significant VIP values were quantitatively analyzed using GC. The test group exhibited significantly higher levels of ethyl acetate and isoamyl acetate compared to the control group (*p* < 0.05). Specifically, the concentration of ethyl acetate in the test group was elevated by 949.94 mg/L compared to the control group. Ethyl acetate and isoamyl acetate levels in the test group were significantly higher than those in the control group (*p* < 0.05), with ethyl acetate showing a 2.09-fold increase compared to the control group (Figure 4A). In addition to esters, alcohols also contributed significantly to the aroma profile of XB, with suitable alcohols enhancing its overall harmony. The experimental group showed significantly lower levels of higher alcohols to the control group (*p* < 0.05), and the contents of isobutanol and isoamyl alcohol decreased by 34.81 mg/L and 23.62 mg/L, respectively (Figure 4B). Meanwhile, the levels of acetaldehyde and acetal in the experimental group were significantly higher compared to those in the control group (*p* < 0.05) (Figure 4C).

### 3.4. Alterations in the Composition of Fungal Communities in Fermented Grains During the Fermentation Process

**Figure 5:**
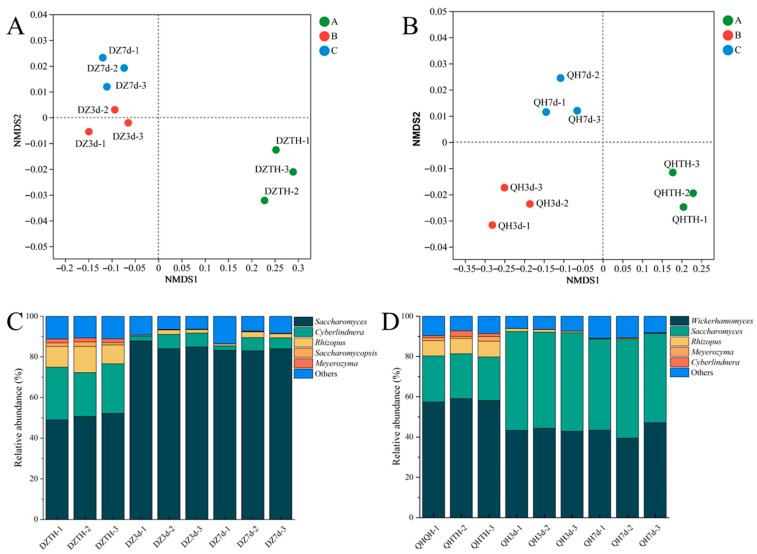
The fungal community structure in fermented grains during the fermentation process. ((**A**) Non-metric multidimensional scaling (NMDS) analysis based on genus level of the fungal community of control group samples. (**B**) Non-metric multidimensional scaling (NMDS) analysis based on genus level of the fungal community of experimental group samples. (**C**) Bar chart based on genus level of the fungal community of control group samples. (**D**) Bar chart based on genus level of the fungal community of experimental group samples).

Non-metric multidimensional scaling (NMDS) analysis using the Bray–Curtis dissimilarity matrix at the genus level was conducted to assess variations in microbial community composition in fermented grains. Significant differences in fungal communities were observed over the course of fermentation (Figure 5A,B). Specifically, at days three and seven of fermentation, samples from the experimental group exhibited greater spatial separation from each other, indicating that intensive WF fermentation influenced the succession of fungal community structure. Bar charts were constructed to visually track changes in fungal community structure in fermented grains (Figure 5C,D). In the fermented grains of the control group, five prominent fungi (with relative abundance > 1%) were identified, namely, *Saccharomyces*, *Cyberlindnera*, *Rhizopus*, *Saccharomymycopsis*, and *Meyerozyma*. Following saccharification, *Cyberlindnera*, *Rhizopus*, *Saccharomymycopsis*, and *Meyerozyma* exhibited the highest relative abundances. However, their levels gradually declined throughout fermentation due to changes in the environment of the fermented grains. *Saccharomyces*, initially at its lowest relative abundance post-saccharification, peaked at day three with an average relative abundance of 90.67%. Subsequently, its relative abundance decreased by day seven, showing a pattern of initial increase followed by a decline over the fermentation period.

Five primary fungi were identified in the fermented grains of the test group, namely, *Wickerhamomyces*, *Saccharomyces*, *Rhizopus*, *Meyerozyma*, and *Cyberlindnera*. Following saccharification, *Saccharomyces*, *Cyberlindnera*, *Rhizopus*, and *Meyerozyma* remained dominant in both the test and control groups, while *Wickerhamomyces* replaced *Saccharomymycopsis* as a dominant fungus in the test group. *Wickerhamomyces* exhibited the highest relative abundance immediately after saccharification, gradually declining throughout fermentation. *Saccharomyces* exhibited an initial increase followed by a decrease, mirroring the trend observed in the control group. *Rhizopus*, *Meyerozyma*, and *Cyberlindnera* maintained lower abundances compared to the control group throughout fermentation. These findings indicate that intensive WF fermentation altered the fungal community structure of the fermented grains.

### 3.5. Metabolic Analysis of the Different Flavor Compounds

**Figure 6:**
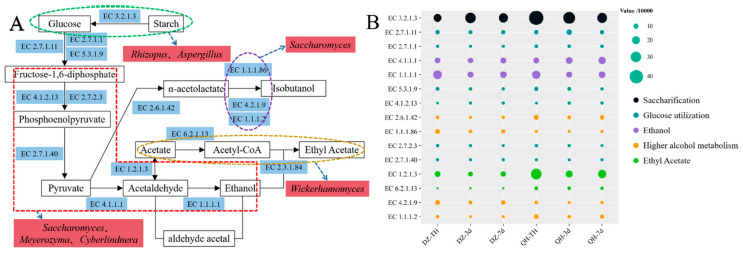
Analysis of fungal metabolic pathways ((**A**) Metabolism involved in microorganisms present in fermented grains. (**B**) The abundance of enzymes).

A metabolic map was constructed based on the annotated fungal species found in fermented grains, the KEGG database, and the significant differences in flavor compounds between the control and experimental groups (Figure 6A). The primary metabolic pathways involving the fungi in the fermented grains included saccharification, glycolysis, ethanol synthesis, higher alcohols synthesis, and ethyl acetate synthesis. Additionally, gene sequences were tagged with PICRUSt to predict the functional enzyme abundance in both the control and test groups, and a bubble map was created (Figure 6B). The relative abundance of EC3.2.1.3 (amyloglucosidase) was significantly higher (*p* < 0.05) in the test group at TH, 3d, and 7d compared to the control group. This enzyme hydrolyzed various sugar-containing compounds in both endo- and exo-cut manners, breaking down starch into sugars usable by microorganisms. Consequently, the residual starch content in the experimental group was significantly lower than in the control group after fermentation (*p* < 0.05), likely due to the increased expression of EC3.2.1.3. The key enzymes involved in glycolytic metabolism included EC2.7.1.1 (hexokinase), EC2.7.1.11 (6-phosphofructokinase), EC5.3.1.9 (glucose-6-phosphate isomerase), EC4.1.2.13 (fructose-bisphosphate aldolase), EC2.7.2.3 (phosphoglycerate kinase), and EC2.7.1.40 (pyruvate kinase). The relative abundance of these enzymes showed no notable difference between the control and experimental groups. EC4.1.1.1 (pyruvate kinase) and EC1.1.1.1 (alcohol dehydrogenase), which are critical for ethanol synthesis, also exhibited no significant difference in relative abundance between the two groups. This lack of difference in the abundance of pyruvate kinase and alcohol dehydrogenase likely explains why the Baijiu yield did not significantly differ between the control and experimental groups.

## 4. Discussion

*Xiaqu Baijiu* is a type of traditional *Baijiu*, which is widely popular for its unique flavor style and high value for money. Previous research indicated that *XQBJ* mainly features volatile aromatic flavor, and ester compounds are the main contributors to its aroma [2,18]. The ester compounds detected in the small-fermented liquor variety are over 20 types, mainly including ethyl acetate, ethyl lactate, ethyl isovalerate, ethyl valerate, etc. Ethyl acetate, the most important aroma compound in *XQBJ*, contributes distinct pineapple and apple notes to the beverage [19]. The content of ethyl acetate is also the highest among the flavor components of *XQBJ*, and reaches over 100 mg/100 mL in high-quality *XQBJ* [3,9]. The concentration of isoamyl acetate in XB was lower, yet due to its lower aroma threshold, it imparted a robust fruity aroma to XB. In the test group, isoamyl acetate increased by 1.63 mg/L, contributing a distinct banana flavor to the beverage. Conversely, the levels of ethyl caprylate and ethyl decanoate decreased, compared to the control group. Esters constituted the primary aromatic compounds in *XQBJ*, and their formation depended on the physicochemical properties of fermented grains and the fermentation environment. At present, there are two methods to enhance the flavor substances of *XQBJ*. One is to modify the process and also to change the fermentation environmental conditions. This approach has been fully explored and utilized [9,20]. The other method is to enhance the fermentation through the use of a functional strain. Non-*Saccharomyces* yeasts are important aroma-producing strains and are widely used in the fermentation of rice wine, fruit wine, and distilled soju [7,11,21]. In this study, *Wickerhamomyces anomalus* was selected and applied in fermented grains of *XQBJ* by *Fuqu*; the content of ethyl acetate increased by 949.94 mg/L, OAVs increased by about 30, and the fruity and floral aromas of *XQBJ* were enhanced. All of these indicate that the selected intensified fermentation strains and methods in this study can increase the main flavor compounds of *XQBJ* and improve the quality of *XQBJ*.

Previous research indicated that *Wickerhamomyces* exhibited greater flavor-compound-producing capabilities compared to *Saccharomyces*, especially in the production of ethyl acetate [8,10]. In this study, the changes in the ethyl acetate content of the *XQBJ* and the abundance of *Wickerhamomyces* showed a positive correlation. The observed increase in ethyl acetate in the fermented grains of the test group could be attributed to the metabolic activity of *Wickerhamomyces*. Changes in the concentration of reducing sugars in the fermented grains partly reflected the balanced and coordinated interaction between saccharification and fermentation rates [1]. At the end of saccharification, the hydrolysis of starch by amylase resulted in a peak in reducing sugars. At this stage, the yeast population was low, and reducing sugars remained high. As fermentation progressed, the yeast and other microorganisms proliferated, utilizing reducing sugars as a carbon source. The levels of reducing sugar decreased quickly and eventually reached a stable state. During the initial three days, which are crucial for *XQBJ* fermentation, the reducing sugar content in the fermented grains of the test group was significantly lower than that of the control group at both 1d and 3d. Despite this, the raw material yield between the control and experimental groups did not show significant differences. This suggests that intensive WF fermentation increased the conversion rate of ethanol without impacting its overall yield. The starch content served as the foundation for alcoholic fermentation, ensuring that the rates of saccharification and fermentation remained balanced to support the normal progression of fermentation in grains. Microorganisms aside from *Saccharomyces* can generate biological enzymes during the brewing process. They utilize fermentation substrates to produce aroma compounds such as esters and higher alcohols, enhancing flavor complexity and thereby elevating the quality of *Baijiu* [22]. Variations in starch conversion yield in liquor may stem from *Wickerhamomyces* metabolism and its interaction with other microorganisms during fermentation, leading to the conversion of starch into diverse flavor compounds, including ethyl acetate, ethyl isovalerate, ethyl decanoate, ethyl octanoate, and isoamyl acetate, etc.

In this study, *Wickerhamomyces* was effectively utilized in the brewing of XB using *Fuqu* as the medium. This approach enhanced the acidity of the fermented grains, improved the starch utilization rate, increased the ethyl acetate content, reduced the higher alcohol content, and overall improved the quality of XB to some extent. Notably, the acidity of the fermented grains increased. The acids in the fermented grains are mainly organic, including acetic acid, propionic acid, butyric acid, lactic acid, amino acids, and higher fatty acids produced during fermentation [23]. The metabolism of these organic acids chiefly involves the glycolysis pathway and the tricarboxylic acid cycle (TCA cycle), with acetic acid being a metabolite of *Wickerhamomyces* and a precursor for ethyl acetate synthesis. The synthesis of ethyl acetate in *Wickerhamomyces* is facilitated by alcohol acyltransferase [10]. Studies have shown that introducing ester-producing functional bacteria into Maotai-flavor liquor significantly increases the acidity of fermented grains [24]. Therefore, it is likely that *Wickerhamomyces* enhances ethyl acetate synthesis by regulating the acidity of the fermented grains and increasing the expression of key enzymes. Additionally, this intensified fermentation alters the fungal community composition in the fermented grains, with *Wickerhamomyces* replacing the dominant fungus *Saccharomymycopsis* in the control group. This is attributed to the significant competitive edge of *Wickerhamomyces* in the fermentation environment, characterized by its strong adaptability to low pH and high ethanol concentrations [25]. This adaptability allows it to quickly dominate the fermentation process. By competing for resources and occupying space, *Wickerhamomyces* further suppresses the reproduction of other yeasts. In the early stages of fermentation, this yeast rapidly proliferates, occupies most of the space in the fermentation system, and consumes a substantial amount of nutrients, such as glucose and amino acids, thereby depriving other yeasts of the resources needed for growth and metabolism [26]. By the end of fermentation, *Wickerhamomyces* could be attributed to increasing ethyl acetate content and decreasing the levels of higher alcohols. Ethyl acetate, an important ester compound, imparts Baijiu with a fresh fruity aroma and pleasant taste. During yeast metabolism, ethyl acetate is largely formed through the action of acetyltransferase, catalyzing acetyl coenzyme A and ethanol. Cordente highlighted that the expression levels of acetyltransferase genes (such as ATF1 and ATF2) directly influence ethyl acetate production [27], and suggested that yeast metabolic pathways, including those regulating fatty acid synthesis, significantly affect ester production [28]. The synthesis of higher alcohols in yeast mainly involves nitrogen source metabolism and the amino acid metabolic pathways. According to Liu, the expression of genes like BAT2, encoding branched-chain amino acid transaminase, plays a vital role in higher alcohols production [29]. They noted that inhibiting the expression of this gene significantly reduces higher alcohols yield. The expression of genes in microorganisms is intricately linked to environmental factors and microbial interactions, which collectively influence the production and functional outcomes of their metabolites. This research offers insights into enhancing the quality of *XQBJ* through augmented fermentation with *Wickerhamomyces*. However, further exploration is needed into the interactions between yeast and other microorganisms in fermented grains.

## 5. Conclusions

This study confirmed that *Wickerhamomyces* plays a critical role in enhancing the fermentation process of XB. Intensified fermentation led to increased acidity in the fermented grains, improved starch utilization, and altered the fungal community composition during fermentation. Additionally, *Wickerhamomyces*-intensified fermentation resulted in higher levels of ethyl acetate and decreased levels of higher alcohols. These findings indicate that *Wickerhamomyces* contributes positively to enhancing the flavor profile of *XQBJ*. Overall, this study provides a method for increasing the content of the main aroma substances in XQBJ, and the results hold promise for enhancing the aroma of *XQBJ*.

## Figures and Tables

**Table 1:** Odor activity value (OAV) and flavor dilution (FD) of esters in *Xiaoqu Baijiu*.

Flavor	Odor	Average Content (mg/L)			Threshold (μg/L)	OAV			FD		
		XQ1	XQ2	XQ3		XQ1	XQ2	XQ3	XQ1	XQ2	XQ3
Ethyl acetate	Floral/fruity	1362.5 ± 235.1	872.3 ± 112.5	1022.7 ± 289.4	32,551.6 ^a^	41.8	26.8	31.4	32	8	16
Ethyl isovalerate	Apple/pineapple	1.8 ± 1.3	2.1 ± 1.6	1.9 ± 0.9	16.9 ^a^	108.4	123.5	112.9	64	64	64
Ethyl caproate	Pineapple	16.2 ± 1.8	15.6 ± 1.4	15.7 ± 2.3	210.4 ^a^	78.3	74.0	74.7	32	32	32
Ethyl octanoate	Apple/pineapple	8.5 ± 0.8	8.8 ± 1.2	9.0 ± 0.8	112.9 ^a^	76.1	77.9	79.4	32	32	32
Ethyl decanoate	Coconut	9.3 ± 1.1	8.7 ± 2.6	8.5 ± 3.2	1122.3 ^a^	8.3	7.8	7.6	4	4	4
Isoamyl acetate	Banana	6.3 ± 1.4	4.8 ± 0.8	4.7 ± 1.7	93.93 ^a^	67.3	51.7	50.3	32	16	16

^a^ Odor thresholds taken from a paper [15].

## Data Availability

The original contributions presented in the study are included in the article/Supplementary Materials. Further inquiries can be directed to the corresponding authors.

## Supplementary Materials

The following supporting information can be downloaded at: https://www.mdpi.com/article/10.3390/foods14193365/s1, Figure S1: Production process of *Xiaoqu Baijiu* (A) and *Fuqu* (B).
