# Supplementary figures and images for: Effect of Intensified Fermentation with Wickerhamomyces anomalus on Fungal Community Structure of Fermented Grains and Flavor Compounds of Xiaoqu Baijiu
Source: Foods. 2025 Sep 29;14(19):3365. doi: 10.3390/foods14193365 (PMC12524076; doi:10.3390/foods14193365)

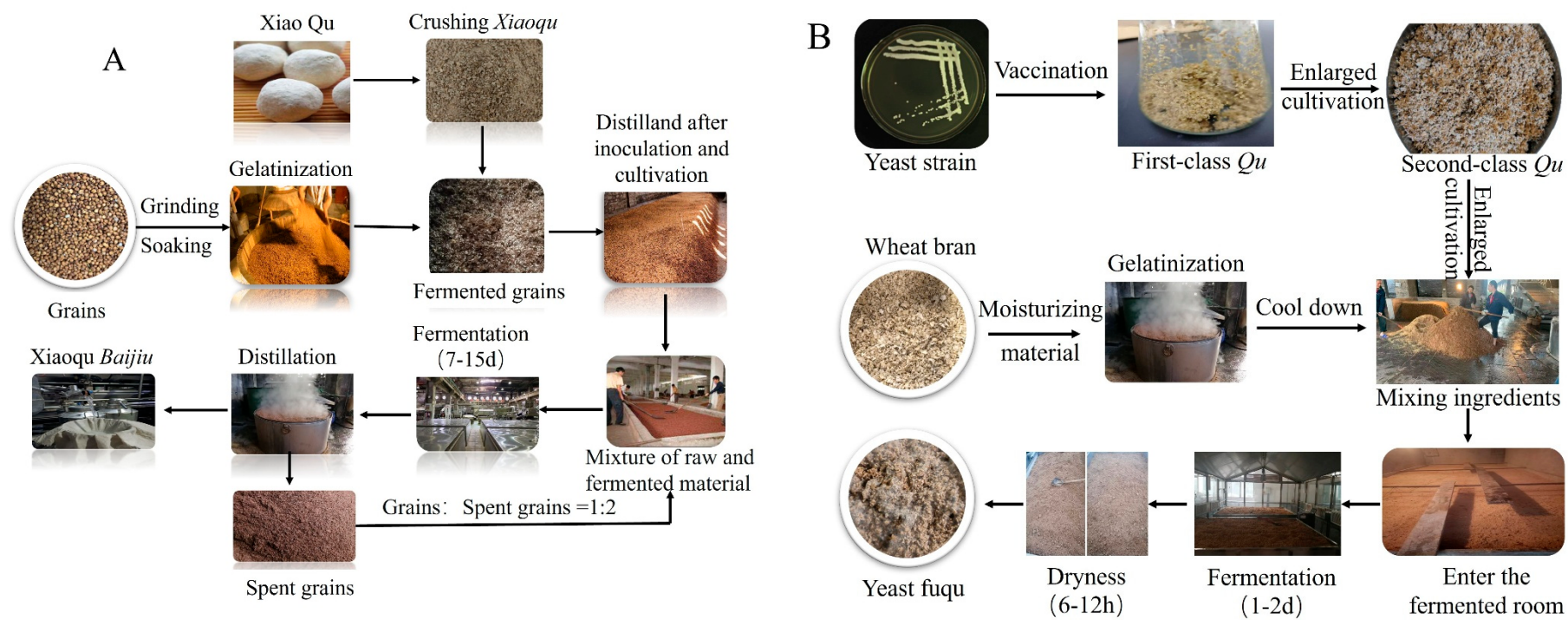

Figure S1 Production process of Xiaoqu Baijiu (A) and *Fuqu* (B)

Supplement: Supplementary file 1 [file foods-14-03365-s001.zip › foods-3858578-supplementary.pdf]
